# Supplementary figures and images for: FTH1 indicates poor prognosis and promotes metastasis in head and neck squamous cell carcinoma
Source: PeerJ. 2023 Nov 17;11:e16493. doi: 10.7717/peerj.16493 (PMC10658887; doi:10.7717/peerj.16493)

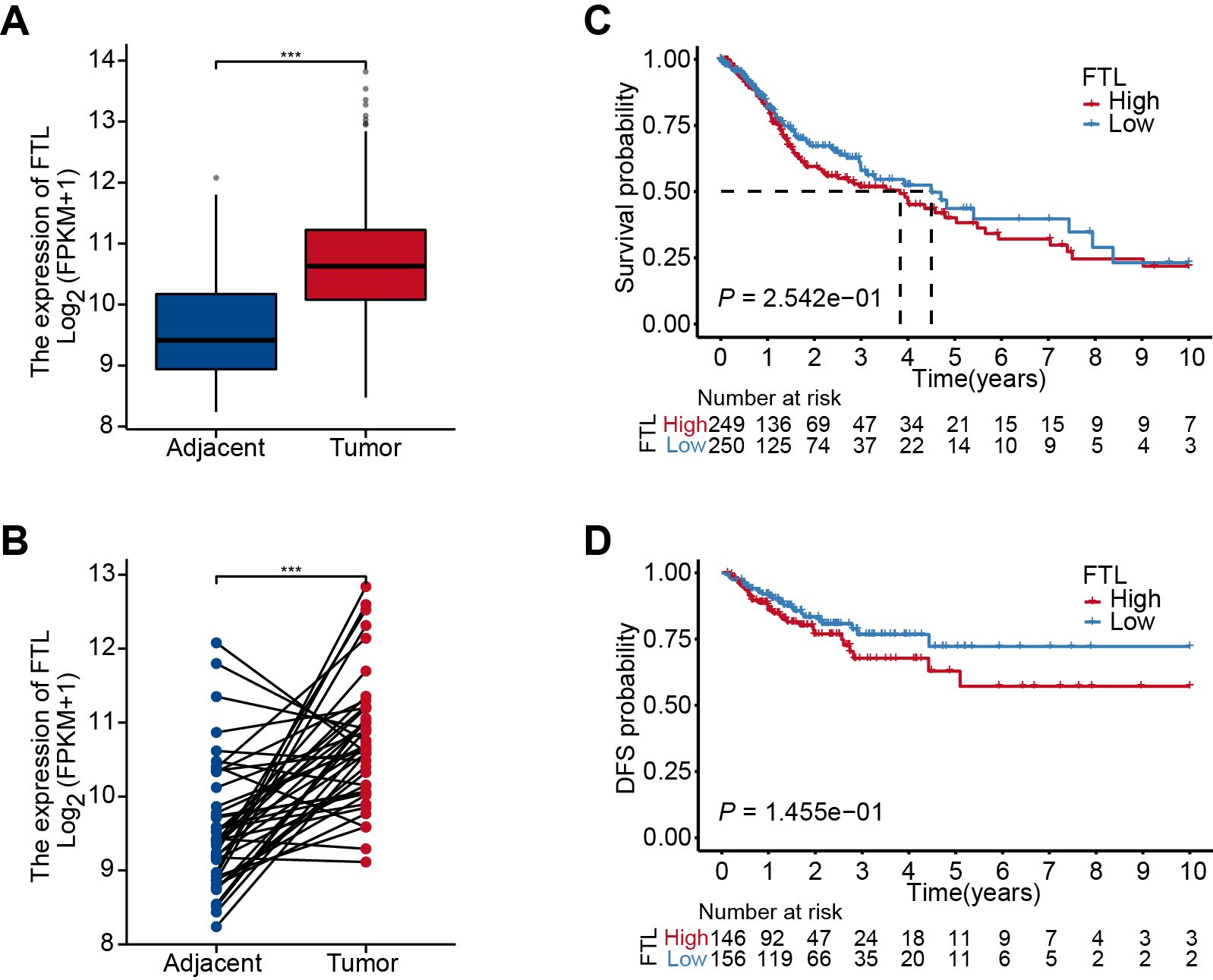

Supplement: Supplemental Information 1 — (A) Differential expression of FTL in HNSCC and adjacent normal tissues in TCGA dataset. (B) Paired difference analysis of FTL mRNA expression in TCGA-HNSCC dataset. Kaplan–Meier curves showed OS (C) and FDS (D) in HNSCC patients in terms of FTL expression. [file peerj-11-16493-s001.png]

WB original data for Figure 2C

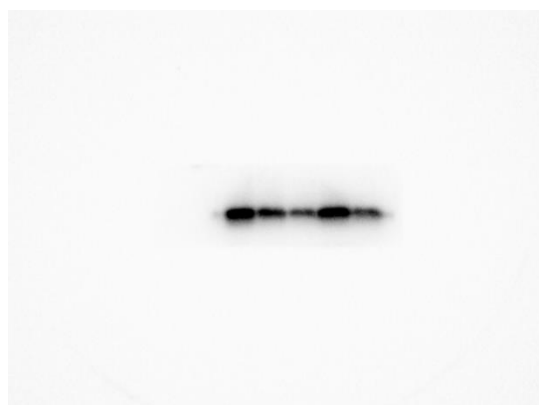

← FTH1

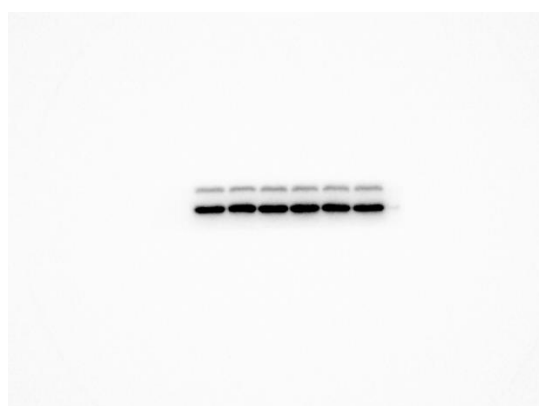

← Actin

WB original data for Figure 9A

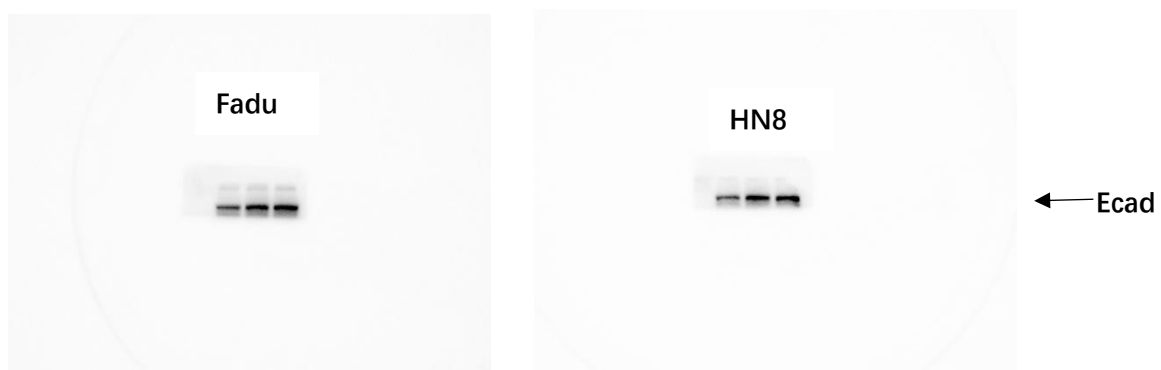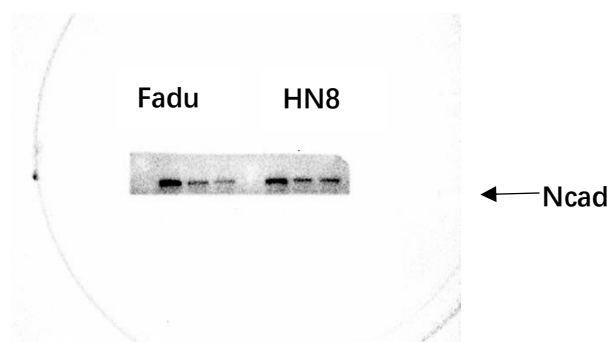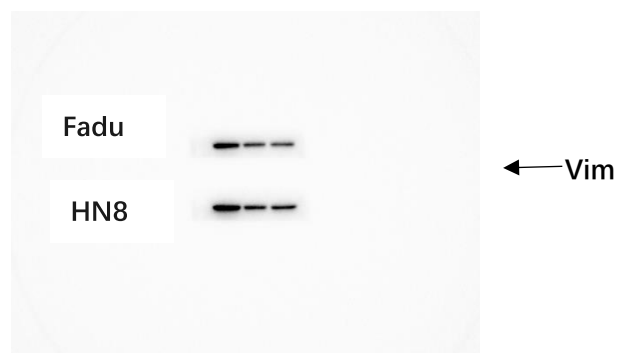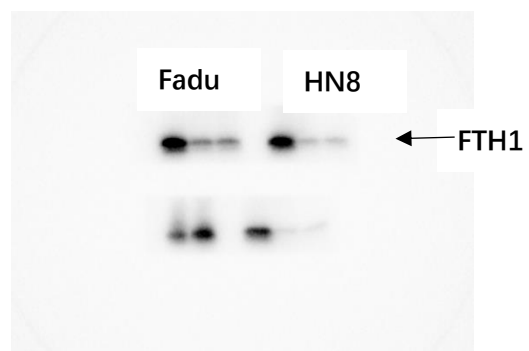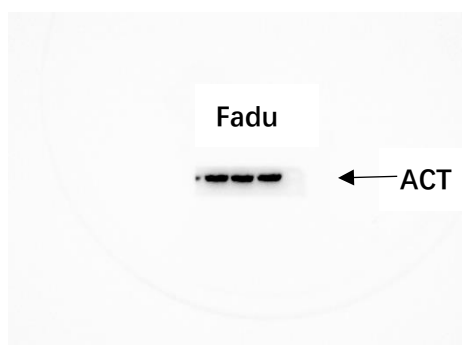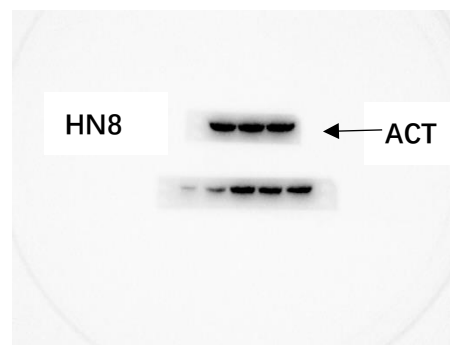

Supplement: Supplemental Information 3 [file peerj-11-16493-s003.pdf]
